# Comparing Administration of Questionnaires via the Internet to Pen-and-Paper in Patients with Heart Failure: Randomized Controlled Trial

**DOI:** 10.2196/jmir.1106

**Published:** 2009-02-06

**Authors:** Robert C Wu, Kevin Thorpe, Heather Ross, Vaska Micevski, Christine Marquez, Sharon E Straus

**Affiliations:** ^6^School of NursingRyerson UniversityTorontoONCanada; ^5^Division of CardiologyUniversity Health NetworkTorontoONCanada; ^4^Department of Public Health SciencesFaculty of MedicineUniversity of TorontoTorontoONCanada; ^3^Knowledge Translation ProgramLi Ka Shing Knowledge InstituteTorontoONCanada; ^2^Division of General Internal MedicineUniversity Health NetworkTorontoONCanada; ^1^Department of MedicineUniversity of TorontoTorontoONCanada

**Keywords:** Heart failure, Internet, questionnaires, validation

## Abstract

**Background:**

The use of the Internet to administer questionnaires has many potential advantages over the use of pen-and-paper administration. Yet it is important to validate Internet administration, as most questionnaires were initially developed and validated for pen-and-paper delivery. While some have been validated for use over the Internet, these questionnaires have predominately been used amongst the healthy general population. To date, information is lacking on the validity of questionnaires administered over the Internet in patients with chronic diseases such as heart failure.

**Objectives:**

To determine the validity of three heart failure questionnaires administered over the Internet compared to pen-and-paper administration in patients with heart failure.

**Methods:**

We conducted a prospective randomized study using test-retest design comparing administration via the Internet to pen-and-paper administration for three heart failure questionnaires provided to patients recruited from a heart failure clinic in Toronto, Ontario, Canada: the Kansas City Cardiomyopathy Questionnaire (KCCQ), the Minnesota Living with Heart Failure Questionnaire (MLHFQ), and the Self-Care Heart Failure Index (SCHFI).

**Results:**

Of the 58 subjects enrolled, 34 completed all three questionnaires. The mean difference and confidence intervals for the summary scores of the KCCQ, MLHFQ, and SCHFI were 1.2 (CI -1.5 to 4.0, scale from 0 to 100), 4.0 (CI -1.98 to 10.04, scale from 0 to 105), and 10.1 (CI 1.18 to 19.07, scale from 66.7 to 300), respectively.

**Conclusions:**

Internet administration of the KCCQ appears to be equivalent to pen-and-paper administration. For the MLHFQ and SCHFI, we were unable to demonstrate equivalence. Further research is necessary to determine if the administration methods are equivalent for these instruments.

## Introduction

Using the Internet as a means to help manage patients with heart failure may improve quality of life and reduce health care costs [1-5]. It is important to further evaluate Internet-based disease management, and its evaluation may be facilitated by using the Internet to administer questionnaires. Indeed, Internet questionnaire administration may have advantages over pen-and-paper administration including being easier for participants to complete, improving completeness of data, and eliminating data entry errors that occur with the transcription of paper questionnaires [6,7]. However, it is important to consider that most questionnaires have been developed and validated for pen-and-paper administration, and there may be important differences between pen-and-paper administration and Internet administration that can affect data quality [8]. Responses of Internet questionnaires may differ from pen-and-paper questionnaires due to issues such as a participant’s computer anxiety or differences in display on a participant’s computer [9]. Whether Internet administration provides similarly valid results as the traditional administration of questionnaires should not be assumed, and it has been recommended that each questionnaire be validated for Internet administration [7,10].

There is some data on the administration of questionnaires via the Internet compared to pen-and-paper administration. Overall, the data implies that Internet administration of questionnaires is associated with lower completion rates but less missing data compared to traditional administration of questionnaires [6]. There is also some evidence concerning whether Internet administration provides similar participant responses as pen-and-paper administration. They appear to be equivalent based on quality-of-life measures in adolescents, health-related questionnaires completed by Internet volunteers, and trauma survey in healthy college volunteers [11-13]. However, to date, there is little data on the equivalence of responses in patients with heart failure or other chronic complex medical conditions. Patients with heart failure differ from patients in previous survey samples by being older and by having more co-morbidities [14].

Since improving quality of life is recognized as one of the main goals of managing heart failure [15], validating questionnaires which assess this parameter is important. We hypothesized that Internet administration would provide similar results as pen-and-paper administration in a cohort of patients with heart failure. We tested this by evaluating equivalency using the test-retest study design of three heart failure questionnaires.

## Methods

### Study Design

This was a prospective trial comparing pen-and-paper administration to Internet administration using classic test-retest design. Between June 2006 and May 2007, we randomized participants to first complete either the pen-and-paper questionnaire or the Internet questionnaire. We then retested the participants two weeks later with the alternate method of administration. The interval of two weeks between retesting was considered short enough to minimize clinical change yet long enough to reduce recall bias.

### Participants

We enrolled patients from the Heart Function Clinic at Toronto General Hospital, University Health Network, Toronto, Ontario, Canada. The Heart Function Clinic is a tertiary care, multidisciplinary heart failure clinic. Patients were eligible for inclusion in the study if they were diagnosed with heart failure, aged 18 years or older, able to access the Internet, able to read and comprehend English, and able to provide informed consent. Participants were given information describing the study at the time of their clinic appointment. For those people interested in participating, the research associate initiated the process of informed consent. Ethics approval was obtained from the research ethics board at the University Health Network. Since this trial did not have an intervention, it was not registered with a randomized trial registry.

### Randomization and Allocation

A computer-generated randomized schedule was prepared by the study biostatistician and then stored and securely concealed until allocation was assigned. For those patients meeting the inclusion criteria and providing informed consent, the research assistant assigned the next randomization sequence according to the schedule. Patients were first allocated to either pen-and-paper or Internet administration. Neither blinding of participants nor the research assistant was possible due to the study design.

### Data Collection

If the participant was randomized to the pen-and-paper version first, they either completed the questionnaires in the clinic or completed them at home and then returned them by mail. If they were initially randomized to the Internet version, they either completed them online at a computer in the clinic or at home. Two weeks later, participants were retested by the alternate method. After one week, email reminders were sent to any participants who had not completed either set of testing.

### Instruments

We administered the following surveys, none of which had been previously validated for use on the Internet:

- Kansas City Cardiomyopathy Questionnaire (KCCQ) [16]. The KCCQ consists of 23 items measuring the impact of heart failure. Including the overall summary score, there are 10 summary scores measuring the dimensions of a patient’s physical function, symptoms, social limitation, self-efficacy, and quality of life. The overall summary score ranges from 0 to 100 with higher scores representing better quality of life. The KCCQ has been validated, used in large randomized controlled trials, and found to be highly responsive [16-19]. A change of over 5 points on the KCCQ summary score is considered to be a clinically significant change in heart failure status [17].

- Minnesota Living with Heart Failure Questionnaire (MLHFQ) [20]. The MLHFQ is a questionnaire that provides a patient’s self-assessment of how heart failure affects his or her daily life. It consists of 21 items, each with the same 0 - 5 Likert scale. The range of scores is 0 - 105 with higher scores representing worse quality of life. Subscores include physical and emotional dimensions. The MLHFQ has been validated and is commonly used as a measure of health-related quality of life of heart failure patients in large randomized controlled trials [21-23]. The minimal clinically important difference is considered to be 5 - 7 points on the total score [24,25].
- Self-Care of Heart Failure Index (SCHFI) [26]. The SCHFI is a 15-item questionnaire measuring self-care. It consists of three subscales: management, maintenance, and self-confidence. The range of scores for each subscale is 16.7 - 100, 25 - 100, and 25 - 100, respectively, with higher scores representing more self-care. The range of the summary scale is 66.7 - 300. While it has been validated, the minimal clinically significant difference is not yet known [26].

### Outcomes

Our primary outcome of interest was the difference in scores between Internet and pen-and-paper administration of the main summary scores for each of the three instruments. Outcomes of secondary interest were the differences in the subscores of the three questionnaires and also whether the order of administration affected participants’ responses.

### Sample Size

As the KCCQ has been found to be more responsive than the MLHFQ [16], this instrument was used to calculate the sample size. In the original validation of the KCCQ, the authors performed a test-retest validation of the KCCQ over a 3-month period [16]. Over the 3-month period, the mean clinical summary score changed by -2.1 in “stable patients” with a *P*-value of .36. This equated to a standard deviation of 14.2. Assuming that half of the variance observed was true change and half was due to measurement error, the standard deviation of change scores due to measurement error would be 10.01. We assumed that any change in the KCCQ over 5 was significant. Using an alpha error of .05 and beta error of .20, we calculated the desired sample size for an equivalence study to be 35 subjects per group and then assumed two groups, resulting in a total of 70 subjects. In retrospect, this calculation likely overestimated our desired sample size, since the analysis was based on paired differences of a single group [27]. Thus, the true sample size required was 35 subjects. Due to slower than expected enrollment, the study was terminated at one year, before the planned enrollment of all 70 subjects was completed.

### Analysis

Mean paired differences between delivery methods were calculated for the summary scores and subscores for each of the three questionnaires. To determine whether the Internet and pen-and-paper administration methods were equivalent, we calculated one-sided confidence intervals. Since a statistical test for an equivalence hypothesis is statistically equivalent to a pair of one-sided hypothesis tests, one-sided confidence intervals were reported as they can be more informative than *P*-values. Given that an acceptable equivalence margin is not precisely known for most of the scales considered, the confidence interval approach provides more detailed information concerning how close the results are between administration methods [28,29]. If one-sided confidence intervals were less than the minimal clinically important difference, the administration methods were considered equivalent for the KCCQ and MLHFQ. Since the minimal clinically important difference was not known for the SCHFI, the mean paired difference and one-sided confidence intervals were calculated to provide information about equivalence.

To determine whether order of administration affected responses, we performed a *t* test on the paired summary scores and sub-scores.

## Results

**Figure 1:**
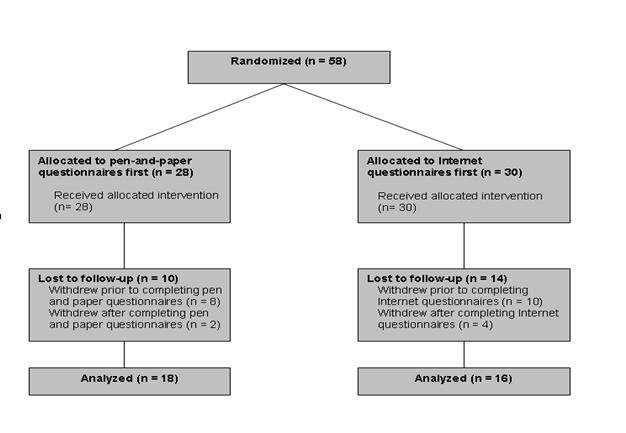
Flow of study participants

From the start of the study in June 2006 until its completion in May 2007, there were a total of 58 participants enrolled. Of these participants, 28 received the paper version first and 30 received the Internet version first (Figure 1). The average age was 51, ranging from 24 to 80 years (Table 1).

**Table 1:** Demographics of participants

		Paper First	Internet First	Completed Both	Total
n		28	30	34	58
Age in Years (SD)		50 (13.3)	52 (15.1)	49 (14.2)	51 (14.2)
Female		7 (25%)	12 (40%)	11 (32%)	19 (33%)
**Highest education achieved**					
	Some High School	0 (0%)	0 (0%)	0 (0%)	0 (0%)
	High School Graduate	7 (25%)	5 (17%)	7 (21%)	12 (21%)
	Some University/College	4 (14%)	4 (13%)	6 (18%)	8 (14%)
	University/College Graduate	14 (50%)	19 (63%)	19 (56%)	33 (57%)
	Post-graduate	1 (4%)	1 (3%)	1 (3%)	2 (3%)
	Undetermined	2 (7%)	1 (3%)	1 (3%)	3 (5%)

There were 34 participants who completed both Internet and pen-and-paper questionnaires. Of these 34 subjects, 18 completed paper questionnaires first, and 16 completed Internet questionnaires first. There were 4 participants who completed Internet questionnaires but did not complete pen-and-paper questionnaires. Conversely, 2 completed pen-and-paper questionnaires but did not complete Internet questionnaires.

The summary scores and subscores for the KCCQ, MLHFQ, and SCHFI are shown in Table 2. For the KCCQ, the one-sided confidence limits of both overall and clinical summary scores were within the equivalence margin of 5, demonstrating that the Internet and pen-and-paper versions are equivalent; for the MLHFQ, the one-sided 95% confidence intervals were larger than the minimally clinical important difference of 5 - 7; and for the SCHFI, there were wide ranges in the one-sided confidence intervals.

**Table 2:** Paired differences and one-sided confidence intervals for the overall and sub-domain scores for the three questionnaires

		Internet			Paper			Difference			95% One-sided CIs	
		n	Mean Score	SD	n	Mean Score	SD	n	Mean Paired Score	SD	Lower	Upper
**KCCQ**												
	Overall Summary Score	38	71.8	19.9	36	70.1	22.0	34	1.2	9.5	-1.5	4.0
	Clinical Summary Score	38	78.0	18.3	36	77.9	19.1	34	-0.1	13.7	-4.1	3.8
	Physical Limitation^a^	37	75.5	23.4	36	75.7	22.6	33	-0.76	21.8	-7.19	5.68
	Symptom Stability	38	54.6	16.3	36	55.6	19.0	34	-0.74	17.9	-5.94	4.47
	Symptom Frequency	38	79.8	19.4	36	78.2	20.9	34	1.96	13.3	-1.91	5.83
	Symptom Burden	38	80.9	19.4	36	81.9	18.4	34	-1.47	13.1	-5.26	2.32
	Total Symptom Score	38	80.4	18.3	36	80.1	19.0	34	0.25	12.0	-3.23	3.72
	Self-Efficacy	38	83.9	17.4	36	82.3	21.6	34	1.10	15.8	-3.48	5.69
	QoL	38	63.8	23.7	36	60.2	27.1	34	2.45	13.1	-1.34	6.24
	Social Limitation^a^	37	66.1	27.2	36	64.3	29.9	33	2.21	13.1	-1.66	6.08
**MLHFQ**												
	Overall	38	39.3	25.6	36	36.4	26.3	34	4.0	20.70	-1.98	10.04
	Physical	38	18.5	11.8	36	15.3	11.5	34	3.8	8.65	1.28	6.31
	Emotional	38	7.1	6.0	36	8.1	6.5	34	-0.88	5.98	-2.62	0.85
**SCHFI**												
	Overall	36	224.2	34.9	34	215.7	31.5	31	10.1	29.4	1.18	19.1
	Maintenance	38	73.4	11.6	36	71.0	12.4	34	2.8	8.3	0.39	5.20
	Management^a^	37	78.0	18.6	34	73.3	16.6	31	5.7	19.2	-0.21	11.5
	Confidence^a^	36	71.4	17.4	35	71.3	18.0	32	1.4	15.6	-3.31	6.05

^a^Note that sample size for some subscores is less than total sample size due to different responses, not due to missing data.

With respect to order of administration, Table 3 summarizes the difference between Internet and pen-and-paper administration for the three questionnaires. The *P*-values were not adjusted for multiple testing. We observed no difference due to the order of administration.

**Table 3:** Effect of order of administration on mean paired differences for the three questionnaires

		Paper First			Internet First			*P* value
		n	Mean Score	SD	n	Mean Score	SD	
**KCCQ**								
	Overall Summary Score	18	4.0	9.1	16	-2.0	9.2	.07
	Clinical Summary Score	18	2.9	12.5	16	-3.5	14.5	.18
**MLHFQ**								
	Overall	18	0.9	14.0	16	7.5	26.4	.38
	Physical	18	3.3	6.3	16	4.3	10.9	.76
	Emotional	18	-2.2	5.5	16	0.6	6.3	.17
**SCHFI**								
	Overall	17^a^	15.8	30.9	14	3.3	26.8	.24
	Maintenance	18	3.3	8.0	16	2.2	8.8	.69
	Management	17 ^a^	7.1	17.2	14	3.9	21.9	.66
	Confidence	18	4.9	19.0	14	-3.1	8.4	.12

^a^Note that sample size for some subscores is less than total sample size due to different responses, not due to missing data.

To determine whether there was a true clinical change over the test-retest interval, we examined the responses to the symptom stability question from the KCCQ: “Compared with 2 weeks ago, have your symptoms of heart failure (shortness of breath, fatigue or ankle swelling) changed?”. Of the respondents, 79% reported no change or no symptoms (n = 27), 15% reported slight changes (n = 5), and 6% (n = 2) reported their symptoms were much better.

## Discussion

### Principal Results

In patients with heart failure, we found that Internet administration was equivalent to pen-and-paper administration for the Kansas City Cardiomyopathy Questionnaire, a questionnaire that is known to be valid and responsive, as well as an independent predictor of poor prognosis [18,19,30].

We were unable to show that Internet administration was equivalent to pen-and-paper administration for the Minnesota Living with Heart Failure Questionnaire and the Self-Care of Heart Failure Index. The MLHFQ was not originally intended to be self-administered; rather, the intention was that research personnel would administer it. This may have affected both pen-and-paper and Internet responses. Indeed, it has been found that who administers the questionnaires (ie, whether self-administered or administered via interview) may have greater effects than how it is administered [8]. While the SCHFI had a larger absolute mean difference and greater confidence intervals than both the MLHFQ and KCCQ, this is likely attributable mostly to the greater range of the summary scale. For the SCHFI, further research to establish the minimal clinically important difference would help to determine if delivery methods are indeed equivalent.

### Limitations

 There were several study limitations of note. Firstly, enrollment was slow and, after one year of recruitment, we did not achieve our desired sample size. While our sample size was much smaller than previous validation studies, this may be due to the fact we studied people with a chronic disease as opposed to people from the healthy population [11-13]. In any case, due to an overestimation of our sample size, we achieved sufficient power to show equivalence for the KCCQ. Secondly, Internet access was a requirement which may have created a biased selection of those who were highly educated and relatively young. Indeed, the average age of our sample was 51 years, much younger than the 72 years which is the average age of patients admitted to our hospital with heart failure [31]. With respect to the level of education, 60% of those enrolled had completed a university or college degree, compared to the 52% possessing the same level of education in the general population of our province [32]. Thirdly, survey completion rate was an issue. Of all who consented and were enrolled in the study, only 58% completed all parts. However, this is similar to other studies comparing pen-and-paper to Internet administration [33]. Finally, we examined three questionnaires but did not randomize the order of the three questionnaires. While our design is similar to other evaluations of Internet questionnaires [13,33], bias may have been introduced because questionnaires that were administered last may be less valid due to participant fatigue. Fatigue increases the chance that participants will provide an answer which is not accurate and may result in a difference in test-retest scores. The order of the questionnaires was as follows: KCCQ, SCHFI, and MLHFQ for paper questionnaires and SCHFI, MLHFQ, and KCCQ for Internet questionnaires. As a result of the order applied, fatigue effects would be least for the SCHFI, moderate for the KCCQ, and most for the MLHFQ. We are reassured by the fact that the KCCQ was still found to be equivalent despite any bias from fatigue.

### Comparison With Prior Work

Previous literature suggests that pen-and-paper administration of questionnaires is equivalent to Internet administration [12,13,33]. To date, these studies have been limited to healthy, younger populations. This study adds to the literature, demonstrating the equivalence between pen-and-paper administration and Internet administration for the KCCQ in patients with heart failure.

### Summary

In summary, Internet administration of the KCCQ appears to be equivalent to pen-and-paper administration. For the MLHFQ and SCHFI, we were unable to demonstrate equivalence, and further research is necessary to determine if the administration methods are equivalent for these instruments.

Our research suggests that one cannot presume equivalency between results from the same questionnaire administered over the Internet and by the pen-and-paper method in individuals with chronic disease. Therefore, it is important that such questionnaires are validated before being used online. Future research should confirm these findings and examine why such differences exist.

## Abbreviations

KCCQ: Kansas City Cardiomyopathy Questionnaire
MLHFQ: Minnesota Living with Heart Failure Questionnaire
SCHFI: Self-Care of Heart Failure Index
